# Acute and chronic demyelinated CNS lesions exhibit opposite elastic properties

**DOI:** 10.1038/s41598-018-37745-7

**Published:** 2019-01-30

**Authors:** Mateusz M. Urbanski, Matthew B. Brendel, Carmen V. Melendez-Vasquez

**Affiliations:** 1 Hunter College, Department of Biological Sciences, New York, NY 10065 USA; 2 Molecular Cytology Core Facility, Zuckerman Research Center, Memorial Sloan Kettering Cancer Center, New York, NY 10065 USA; 3 The Graduate Center of the City University of New York, New York, NY 10016 USA

## Abstract

Increased deposition of extracellular matrix (ECM) is a known inhibitor of axonal regrowth and remyelination. Recent *in vitro* studies have demonstrated that oligodendrocyte differentiation is impacted by the physical properties of the ECM. However, characterization of the mechanical properties of the healthy and injured CNS myelin is challenging, and has largely relied on non-invasive, low-resolution methods. To address this, we have employed atomic force microscopy to perform micro-indentation measurements of demyelinated tissue at cellular scale. Analysis of mouse and human demyelinated brains indicate that acute demyelination results in decreased tissue stiffness that recovers with remyelination; while chronic demyelination is characterized by increased tissue stiffness, which correlates with augmented ECM deposition. Thus, changes in the mechanical properties of the acutely (softer) or chronically (stiffer) demyelinated brain might contribute to differences in their regenerative capacity. Our findings are relevant to the optimization of cell-based therapies aimed at promoting CNS regeneration and remyelination.

## Introduction

Despite the presence of oligodendrocyte progenitor cells (OPC) capable of regenerating myelin after its loss, chronic multiple sclerosis (MS) lesions in the brain and the spinal cord are characterized by remyelination failure. Cell-based therapies involving progenitors derived from autologous stem cells are a promising approach for the treatment of chronic MS^1,2^. However, transplants delivered into CNS areas with extensive damage often result in poor cell engraftment and survival^3–5^. To address this issue, attempts have been made to design bio-compatible scaffolds and injectable hydrogels that can be used to fill wound cavities or lesions and provide a matrix supportive of neuronal and glial development^6,7^. Recent studies have also demonstrated that mechanical cues delivered by the extracellular matrix (ECM) are capable, independently of chemical signals, of directing the differentiation of stem cell populations or promoting the differentiation of mesenchymal stem cells into specific cell types. Similarly, while soft artificial substrates mechanically similar to the healthy brain promote neurogenesis and axonal growth^8,9^, work from our laboratory and others has demonstrated that oligodendrocytes (OL), the myelinating glia of the CNS, are also mechanosensitive^10–14^ and that increases in ECM stiffness inhibit their differentiation^14^.

Although the abnormal accumulation of a wide range of ECM proteins in demyelinated lesions has repeatedly been shown to inhibit remyelination^15–17^, less is known about how the ECM changes mechanically during the time course of disease and recovery in the CNS. Although it is accepted that chronic demyelination ultimately causes remyelination failure^18^, and that chronic and acute demyelination are associated with the deposition of different types of ECM^15,16^, there has been no systematic analysis of how demyelinating insults affect the mechanical properties of the ECM in the CNS. More critically, attempts to measure the mechanical properties of the brain parenchyma in animal models of demyelination^19^ and human aging^20^ using non-invasive methods such as magnetic resonance elastography (MRE) have been limited to a macroscopic spatial resolution.

Several studies have analyzed the properties of healthy and pathological brain tissue using MRE. For example, Schregel *et al*.^19^ examined the elastic properties of the mouse corpus callosum during a 12-week course of cuprizone induced demyelination. Their results showed an initial increase in stiffness, followed by a decrease at the 12-week time point associated with remyelination failure and steady increase in ECM deposition. Human MRE studies^21,22^ comparing the brains of healthy volunteers and multiple sclerosis (MS) patients also found a 13–20% reduction in parenchymal stiffness. These findings of decreased mechanical rigidity seem somewhat counterintuitive given the increased deposition of ECM components noted in MS lesions and animal models of demyelination. Although it is possible that ECM deposited as a result of demyelinating insult is not structurally coherent, increased ECM deposition generally correlates with increased tissue stiffness, as seen in injury-induced tissue fibrosis and tumor progression^23–26^.

This apparent contradiction may be explained by the low spatial resolution of the non-invasive MRE approach. For example, the human studies^21,22^ used imaging with a resolution of approximately 1 mm, while in the mouse study^19^, the target demyelinated region was represented by 6–8 pixels at 200 µm resolution. Since many of the ECM aggregates present in demyelinated lesions appear to be of cellular or sub-cellular size^15,27^, it is likely that lower-resolution methods understate or overlook their mechanical contribution.

Atomic force microscopy (AFM) provides an alternative that allows the examination of tissue stiffness at scales relevant to cellular mechanotransduction. To this end we have developed a protocol, which allows us to capture both optical images and AFM measurements of the brain tissue at higher resolution. This information used in combination with immunohistochemistry of the same region, makes it possible to correlate changes in tissue stiffness with ECM structure and cellular composition of the area being examined. This approach provides greater detail on the mechanical properties of the CNS and the variability of mechanical stimuli potentially experienced by cell populations within demyelinated lesions.

We found that acute and chronic demyelination affect the mechanical properties of CNS tissue in distinct ways when examined at cellular rather than macroscopic resolution. Specifically, acute demyelinated lesions are softer than healthy tissue, while chronic demyelinated lesions exhibit increased stiffness, which is associated with elevated ECM deposition. Thus, changes in ECM mechanical properties may be an important contributing factor to the rapid remyelination observed in acute softer lesions and the failure to remyelinate typical of chronic stiffer lesions.

## Results

### Measuring the mechanical properties of the CNS in health and disease: the importance of scale

**Figure 1:**
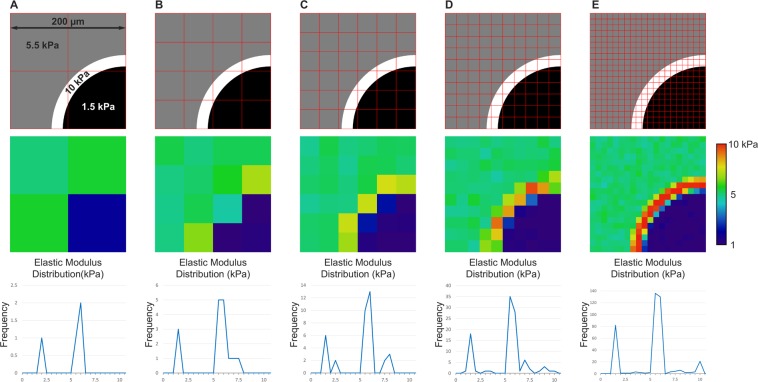
A model of the effects of spatial resolution on the measurement of mechanical properties of pathological tissue. Top panels: Schematic diagram of a sample consisting of three regions (grey, white and black) with different tissue stiffness (1.5, 5 and 10 kPa) within a 4 mm^2^ area (200 × 200 μm). The square grid overlaid in each panel (**A** to **E**) indicates the size of regions individually sampled by AFM. Middle panels: Heat maps of tissue stiffness generated in Matlab comparable to those obtained experimentally in this study, illustrating the exponential increase in resolution, as the size of each individual region sampled decreases. Bottom panels: Histograms showing the shift from a bi-modal distribution to a more accurate tri-modal distribution when stiffness is measured at higher resolution (400 measurements at 1 × 1 μm in **E**).

To better illustrate the effects of spatial resolution on the measurement of mechanical properties of lesioned tissue, we generated a basic model using Matlab. Figure 1 presents an idealized 200 × 200 µm tissue section, containing a lesion with a soft center (1.5 kPa) surrounded by a narrow border of scar tissue (10 kPa) and a large normal-appearing area (5.5 kPa). The effects of a ten-fold increase in sampling resolution on the apparent elastic modulus of the tissue are shown (Fig. 1A–E, top panels). At the lowest resolutions (1A,B) the apparent stiffness distribution is bi-modal, and the impact of the high-stiffness region on average tissue elasticity is undistinguishable from the effects of random variability. Increasing resolution (Fig. 1C,D) allows the higher-stiffness lesion border to be identified. Even at this enhanced level of resolution, the measurements still contain many artifacts, (Fig. 1C bottom panel, peaks at 2.5 and 7.5 kPa) that do not accurately represent the overall mechanical properties of the lesion. Once the resolution reaches the cellular scale (Fig. 1E), it becomes clear that the tissue consists of three well-delineated regions of specific elasticity, and the measured values for all populations begin to match the ideal ones. This model clearly illustrates the exponential increase in detail and the changes in stiffness distribution when measurements are performed at microscopic, cellular resolution.

**Figure 2:**
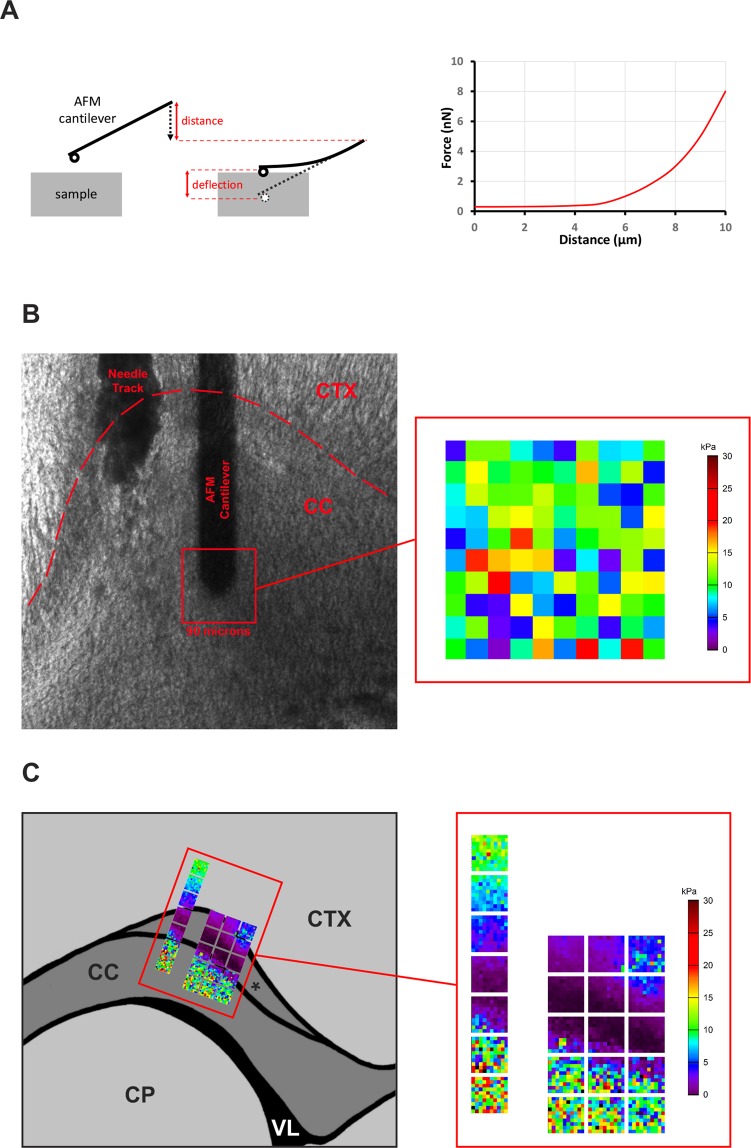
AFM protocol for the mapping of mechanical properties of CNS tissue. (**A**) Schematic representation of an AFM elasticity measurement. The deflection of the cantilever (tipped with a spherical probe) resulting from its contact with the sample is used to derive a force-indentation curve (right panel), with slope proportional to sample elasticity. The force-indentation curves are fitted to the Hertz model for spherical probes^55^ to calculate the Young’s modulus of the sample, expressed in kilopascals (kPa). Multiple measurements performed in a semi-automated manner over a 90 × 90 µm are used to generate heat maps of tissue elasticity, as shown below. (**B**) Brightfield image of a 100 µm coronal section from a lysolecithin-injected brain prepared for AFM measurement. Red square indicates the 90 × 90 µm area being sampled centered on the tip of the AFM cantilever, and the resulting map of tissue elasticity. The cortex (CTX) and corpus callosum (CC) are clearly identified as is the needle track left by the lysolecithin injection. (**C**) Representative force measurements from the cortex and anterior corpus callosum of uninjured contralateral hemisphere. Using anatomical landmarks as reference, larger maps of normal tissue stiffness can be assembled from individual scans. The corpus callosum, cingulum (*) and the adjacent cortical layers are mechanically distinct from each other (CP: caudoputamen, VL: lateral ventricle).

Existing AFM data confirms that most tissues, including the brain, exhibit a significant degree of mechanical heterogeneity when sampled at the microscopic level. For example, the 90 × 90 µm AFM stiffness map in Fig. 2B obtained from a coronal section through the mouse corpus callosum (CC), shows that mechanical properties vary significantly across apparently uniform tissue, while the composite in Fig. 2C makes it evident that a 200 or 300 µm-wide section of tissue can easily contain several mechanically distinct regions. Similar patterns can be observed in other published AFM measurements of the rodent brain, including one characterizing the mechanical properties of the rat hippocampus^28^ as well as an examination of the effects of stab injury on the elastic properties of the mouse cortex and spinal cord^29^.

### An AFM-based protocol for mapping of the mechanical properties of CNS tissue

We set out to develop an experimental protocol for AFM measurement of brain tissue that would provide the high-resolution data necessary to establish how demyelination and remyelination affect tissue stiffness at the microscopic level. While AFM delivers superior spatial resolution, its ability to produce reliable stiffness measurements depends heavily on the preparation of the sample. Specifically, the surface being measured should ideally be extremely uniform, and free of debris which may interfere with the probe cantilever (see Fig. 2A). For example, we found that fresh mouse forebrain thick coronal sections (manually-cut), yielded AFM stiffness values of 1.87 ± 0.87 kPa (mean ± SD) the anterior corpus callosum and adjacent cortex^14^. This value is within the range most commonly cited for brain tissue. However, measurements performed on comparable fresh tissue which had been sectioned (300 μm) with a vibratome resulted in surprisingly low and invariable readings of 0.25 ± 0.05 kPa in the same area of the brain (Table 1), suggesting extensive mechanical damage to the surface of the sample. Due to these observations, we decided to use cryo-sectioned fixed tissue for our demyelination experiments. While this approach yields data on relative changes in elastic properties rather than measuring absolute values, it increases reproducibility and produces thin optically transparent sections. In addition, the greater uniformity of the sectioned surface allows for the use of smaller AFM probes and higher spatial resolution. This allowed us to capture both optical images and AFM “heat maps” of tissue stiffness **(**Fig. 2B,C**)**. Since the fixed sections are intact following the AFM measurements, they can then be processed for immunohistochemistry (IHC) to visualize the state of the myelin and ECM deposition in the mapped areas.

**Table 1:** The effects of sectioning method on the mechanical properties of mouse forebrain tissue.

Tissue Preparation Prior to AFM	Ε ± SD (kPa)
Vibratome cut coronal sections of fresh mouse brain (100 µm)	0.25 ± 0.05
Manually cut coronal sections of fresh frozen mouse brain (~1 mm)	1.9 ± 0.9

Measurements (n = 500 vibratome cut, n = 300 manually cut) were collected from 2 brain sections in each condition.

### Acute lysolecithin-induced demyelination of the mouse corpus callosum results in tissue softening, but chronic lesions exhibit areas of significantly increased stiffness

**Figure 3:**
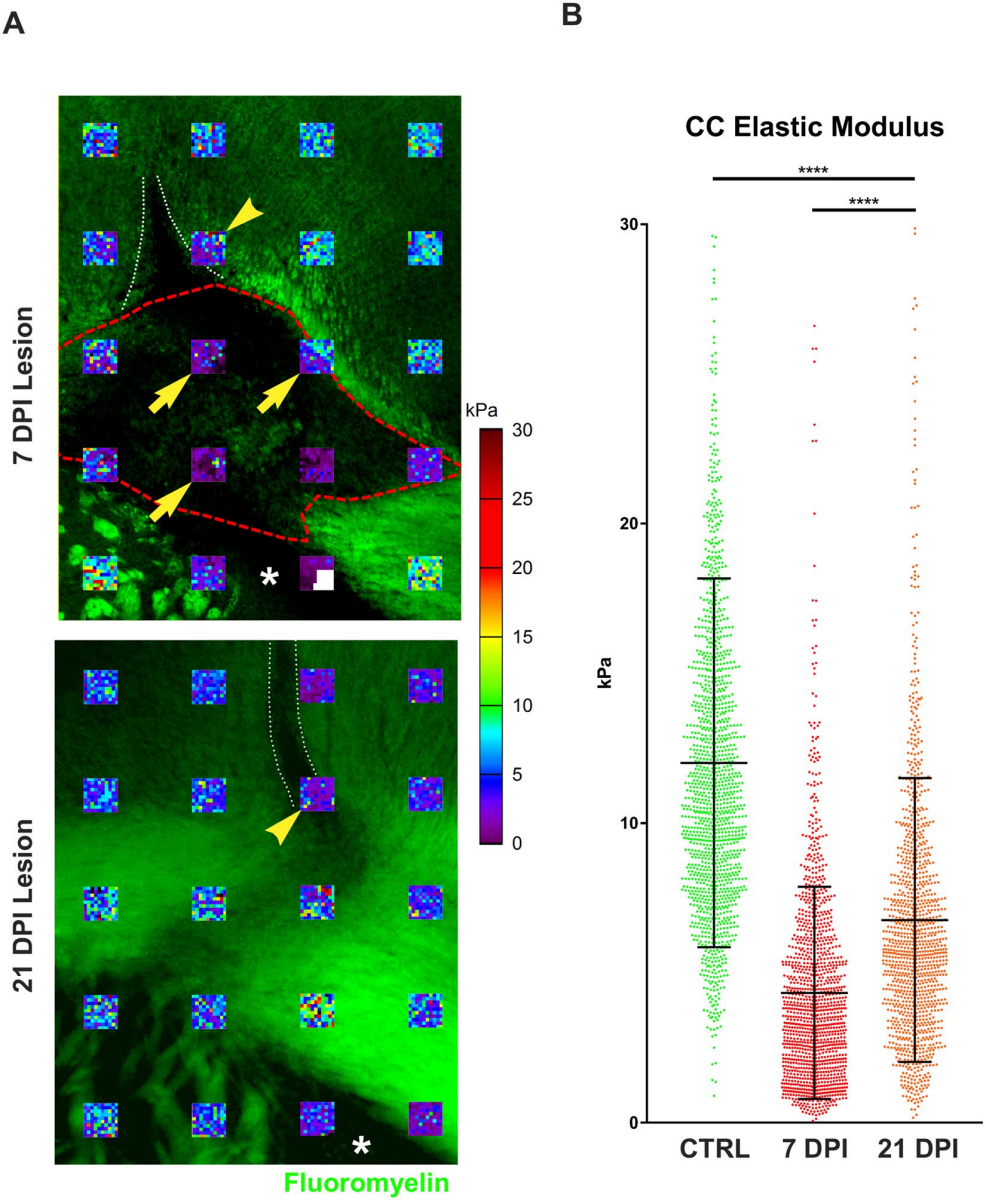
Acute lysolecithin lesions of the mouse corpus callosum are softer than healthy tissue. (**A**) Overlay of 90 × 90 µm AFM stiffness maps and myelin staining (green), demonstrating the relationships between myelin loss and changes in corpus callosum stiffness. Arrows indicate maps within demyelinated lesion while arrow heads indicate maps located in areas containing apparent mechanical damage due to lysolecithin injection (white dotted lines). (**B**) Quantitation of corpus callosum elastic modulus in the lesion areas shown in (**A**) compared with uninjured contralateral tissue (CTRL). A sharp decrease in tissue stiffness at the peak of demyelination (7 days post-injection, 7 DPI) is followed by incomplete recovery after remyelination (21 DPI) and recovery of fluromyelin staining. AFM measurements (n = 1400 for CTRL, n = 1299 for 7 DPI and n = 1199 for 21 DPI) were collected from 2–5 brains per time point. Lines represent the mean ± SD for all measurements; ****p < 0.0001, Mann-Whitney test.

Initial AFM testing performed on acute lesions from the lysolecithin-induced model of demyelination^30^ indicated a dramatic decrease in tissue stiffness (Fig. 3B) 7 days post lysolecithin injection (demyelination peak) compared to the uninjured contralateral corpus callosum (4.34 ± 2.55 vs. 12.01 ± 6.16 kPa; mean ± SD). Following remyelination (21 days post-injection), tissue stiffness partially recovered to 7.15 ± 0.18 kPa. These findings agree with previous data indicating a direct correlation between myelin content and brain stiffness^31^. However, while the areas of decreased stiffness clearly matched to areas of myelin loss as confirmed by loss of fluoromyelin staining at 7 dpi (arrows in Fig. 3A, top panel); local lysolecithin injection also causes tissue trauma at the injection site (white dotted lines in Fig. 3A,B). Of note, a recent study^29^ which used AFM to examine the effects of traumatic stab injury in a similar region of the mouse brain, found extensive softening resulting from mechanical disruption of the tissue. Although we avoided recording measurements in the mechanically damaged areas, it is still possible that some of the sampled regions exhibiting reduced stiffness fall within or close to the needle track (arrowheads in Fig. 3B). We therefore decided to conduct further studies using cuprizone-induced demyelination, as it allows for the modeling of both acute and chronic demyelination in the same CNS area with no possibility of mechanical damage contributing to potential changes in tissue stiffness.

### Acute cuprizone-induced demyelination results in tissue softening followed by recovery, while chronic lesions exhibit areas of increased stiffness and elevated deposition of ECM components

In the cuprizone-induced model of demyelination, mice fed chow containing 0.2% cuprizone experience metabolic stress which results in widespread apoptosis of oligodendrocytes, accompanied by macrophage infiltration, microglia activation and extensive astrogliosis^32^. This leads to widespread demyelination of the corpus callosum, but if the toxin is withdrawn after 6 weeks of treatment, extensive remyelination is possible. Extending the treatment to 12 weeks results in multiple failed cycles of remyelination and, eventually, chronic and irreversible demyelination. Although there is some regional variation in the extent of demyelination^33^, the medial region of the caudal corpus callosum consistently displays complete demyelination in this model, and it is the area we have used for these studies. Appropriate age-matched animals fed normal chow were used as controls, ranging from 17 to 20 weeks of age.

**Figure 4:**
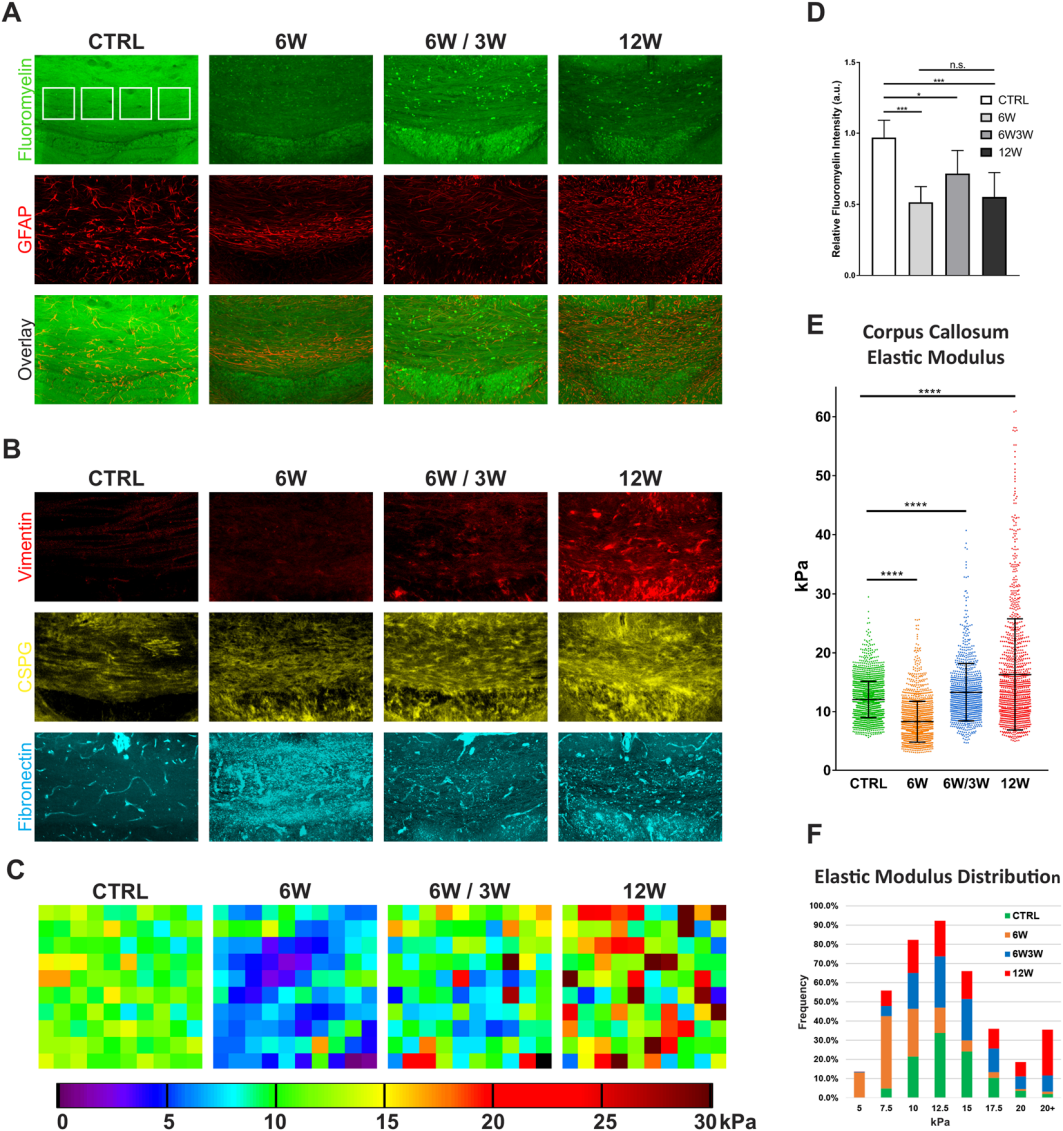
Chronically demyelinated tissue displays a significant increase in stiffness, while acute demyelination leads to decreased tissue stiffness. (**A**) Confocal projections of the medial portion of the caudal corpus callosum (CC) demyelinated by cuprizone (CPZ) treatment, with myelin visualized with fluoromyelin (green) and astrocyte activation by GFAP staining (red). White squares indicate the regions (90 × 90 µm) sampled by AFM within each specimen. (**B**) Representative images of demyelinated CC, showing an increase in the expression of vimentin, chondroitin sulfate proteoglycans (CSPG) and fibronectin after CZP treatment. (**C**) Representative 90 × 90 µm AFM force maps from each of the four conditions depicted above, showing cellular-scale changes in stiffness resulting from acute demyelination (6 W), remyelination (6 W/3 W) and chronic demyelination (12 W). (**D**) Quantitation of fluoromyelin staining in the CC at different stages of CPZ treatment. Values were normalized to cortical fluoromyelin staining. A significant reduction of fluoromyelin staining (***p < 0.001; Mann-Whitney test) was observed after 6 W (0.51 ± 0.11) and 12 W (0.55 ± 0.17) of CPZ treatment when compared to CTRL tissue (0.97 ± 0.12). A partial but significant recovery of fluoromyelin staining (0.71 ± 0.16, *p = 0.01) following remyelination (6 W/3 W) was also observed. Bars represent the mean ± SD, n = 8 sections per condition. (**E**) Quantitation of AFM measurements from the CC. Acutely demyelinated tissue from animals which received 6 weeks of CPZ (6 W) shows a significant decrease in elastic modulus compared to control (CTRL), which recovers to near-normal values after CPZ withdrawal and remyelination (6 W/3 W). By contrast, 12-week CPZ treatment (12 W) and chronic demyelination results in a significant increase in tissue rigidity. Graph represents mean ± SD from AFM measurements (n = 2156 for CTRL, n = 1163 for 6 W, n = 980 for 6 W/3 W and n = 1171 at 12 W) taken from 3–5 animals per condition (****p < 0.0001, Mann-Whitney test). (**F**) Histogram of the stiffness values quantitated in (**E**).

As shown in Fig. 4A (top panels), cuprizone treatment resulted in the expected and well-established pattern of acute demyelination at 6 weeks (6 W), followed by partial remyelination in the 6 weeks of cuprizone and 3 weeks of recovery cohort (6 W/3 W), and remyelination failure in the 12-week group (12 W). This manifested itself as a significant and similar 50% decrease in fluoromyelin staining in both acute and chronic demyelination lesions compared to CTRL tissue, followed by a 20% recovery after remyelination. (Fig. 4D). Similar to our findings in the lysolecithin model, acute cuprizone demyelination resulted in a significant decrease in tissue stiffness when compared to controls values (8.28 ± 3.49 kPa vs. 12.07 ± 3.12 kPa; mean ± SD). This was followed by recovery to near-normal values after cuprizone withdrawal and remyelination (13.3 ± 4.90 kPa). By contrast, a significant increase in overall tissue stiffness (16.32 ± 9.46 kPa) was apparent in animals which had undergone chronic demyelination and failed to remyelinate (Fig. 4E).

This increase in tissue stiffness was accompanied by astrogliosis, as shown by elevated GFAP and vimentin staining (Fig. 4A,B) and accumulation of ECM components such as chondroitin sulfate proteoglycans (CSPG) and fibronectin. Notably, the increased tissue stiffness observed in the 12-week condition was not due to a proportional increase in all areas probed, but rather to a elevated incidence of regions of very high stiffness causing a shift in the overall distribution. Thus, while in the control condition only 2% of the regions measured registered as 20 kPa or higher, in the 12-week condition they accounted for 24% of all measurements (Fig. 4F). In addition, the maximum stiffness value observed increased by two-fold, from 30 kPa in the controls to 61 kPa in the 12-week samples (Fig. 4E), consistent with the formation of dense aggregates containing ECM proteins. These results support our hypothesis that changes in ECM deposition in chronically demyelinated tissue are associated with measurable and significant increase in tissue stiffness at the microscopic scale.

### Increased tissue stiffness in human MS lesions correlates with increased ECM deposition

**Figure 5:**
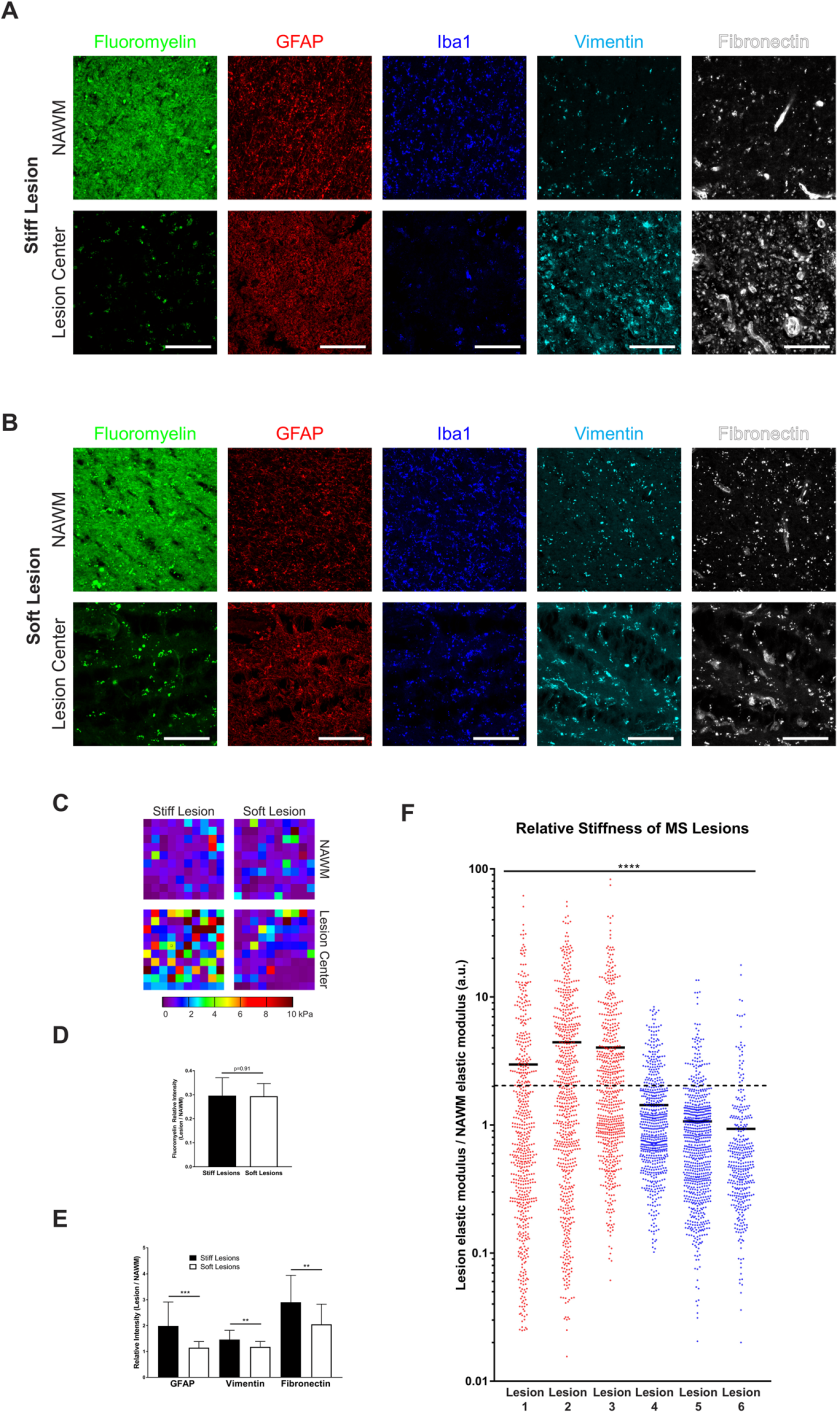
Increased ECM deposition correlates with greater tissue stiffness in human MS lesions. (**A**,**B**) Representative images showing MS lesions (stiff and soft) and adjacent normal-appearing white matter (NAWM). The extent of myelin loss (fluoromyelin), presence of a reactive astrocytes (GFAP, vimentin) and monocyte/microglia infiltration (Iba1), as well as ECM deposition (fibronectin) are shown. (Scale bars 100 µm) (**C**) Representative 90 × 90 µm AFM force maps from the lesions shown in A and B. Significant changes in elasticity are detectable within each lesion compared to adjacent NAWM. (**D**,**E**). Quantitation of relative mean staining intensity for fluoromyelin, GFAP, vimentin and fibronectin normalized to staining intensity within adjacent NAWM. Bars indicate mean ± SD, n = 25 fields for stiff lesions, n = 24 fields for soft lesions, **p < 0.01, ***p < 0.001 Mann-Whitney test). (**F**) Quantitation of AFM stiffness from six individual MS lesions (stiff in red; soft in blue). Values are expressed as lesion stiffness relative to adjacent NAWM. Dotted line indicates two-fold increase in stiffness compared to NAWM. Bars indicate the mean value from all measurements (n = 592, 693, 647, 650, 748 and 384 for lesions 1–6, respectively, ****p < 0.0001, for differences between groups, Kruskal-Wallis test).

To examine whether the changes observed in the animal demyelination models are also detectable in human MS tissue, we performed AFM mapping on six MS lesions from two age and sex-matched samples from patients diagnosed with secondary progressive multiple sclerosis, and whose brains were collected shortly after death (Table 2). Lesions analyzed were located within the forebrain white matter underlying Brodmann’s areas 6 and 8 (Lesions 1, 2 and 4 in sample 441; lesions 3, 5 and 6 in sample 575). The lesions were initially identified by a pathologist and later confirmed by staining with fluoromyelin, GFAP and Iba1 (Fig. 5A,B). In each case, AFM measurements were performed in a grid pattern at 500 µm intervals at multiple points within the lesion, and they were compared to adjacent normal-appearing white matter (NAWM) (Fig. 5C). Additionally, following the AFM measurements, tissue sections were stained for GFAP, vimentin and fibronectin, to evaluate the extent of astrogliosis and ECM deposition within and around the MS lesions.

**Table 2:** Clinical details of the human tissue samples used in the study.

Sample ID	Diagnosis	Age at Death	PMI^*^	Sex
441	Secondary Progressive MS	62	4 hours	F
575	Secondary Progressive MS	64	7 hours	F

*post-mortem interval before tissue collection.

We found that the lesions examined fell into two main categories (Fig. 5): ones with relatively unchanged mechanical properties compared to surrounding NAWM (“soft”) and those with significantly increased elastic modulus (“stiff”). The stiff lesions (lesions 1–3) displayed a 3 to 4 fold increase in elastic modulus over adjacent NAWM (2.97 ± 6.06, 4.44 ± 7.02 and 4.03 ± 7.10; mean ± SD), while soft lesions (lesions 4–6) exhibited 1 to 1.4 fold changes only (1.43 ± 1.41, 1.07 ± 1.39 and 0.93 ± 1.65). Of note, the percentage of regions displaying changes of 2-fold or more above NAWM stiffness was significantly higher in stiffer lesions than in soft lesions (30–46% vs. 9–20%). These findings indicate that the overall increase in tissue stiffness is driven by mechanical changes widely affecting the lesioned area, and not limited to a few outlier regions.

Interestingly, the stiff lesions bear some of the hallmarks of chronic MS lesions, including sharply delineated lesions margins apparent upon pathological examination (data not show) and limited inflammatory response (Iba1 + cells) within the lesion area (Fig. 5A). By contrast, softer lesions presented a morphology consistent with an active lesion, with a diffuse margin, and greater inflammatory response within the lesion area^15^ (Fig. 5B). In addition, the stiff lesions exhibited increased astrogliosis as evidenced by increased GFAP and vimentin staining, as well as greater deposition of vasculature-associated fibronectin (Fig. 5E). Compared to softer lesions, the relative fluorescence intensity of IHC for GFAP was increased by 73% in stiffer lesions (1.99 ± 0.93 vs 1.15 ± 0.24, mean ± SD) and by 24% for vimentin (1.46 ± 0.36 vs 1.18 ± 0.21, mean ± SD), while that of fibronectin by 41% (2.90 ± 1.04 vs 2.05 ± 0.78). However, the relative fluoromyelin intensity at the lesion center (Fig. 5D) was comparable in both conditions (0.30 ± 0.07, stiff vs. 0.29 ± 0.05 soft), further indicating that differences in stiffness between soft and stiff MS lesions are likely driven by changes in glial reactivity and ECM deposition. Of note, initial analysis from a periventricular chronic MS lesion (data not shown) also identified a well-defined stiff lesion border with increased fibronectin deposition, separating the NAWM from the demyelinated soft lesion center. This findings suggests that despite anatomical differences, an increase in stiffness is a consistent feature of augmented ECM deposition and astrogliosis in MS lesions.

Collectively, these results demonstrate similarities in the mechanical properties of stiff human MS lesions and chronic demyelination in the mouse cuprizone model and reveal a consistent pattern of changes in tissue stiffness in both acute and chronic demyelination.

## Discussion

Our data indicate a complex relationship between demyelination and tissue stiffness. Acutely demyelinated lesions, which retain the potential for spontaneous remyelination, display lower stiffness than healthy tissue in both the lysolecithin and cuprizone models. Furthermore, the stiffness values recover to near-normal levels over the course of remyelination. Conversely, tissue from the cuprizone chronic demyelination model, which has experienced several failed cycles of remyelination, is stiffer than healthy white matter. Since increased matrix stiffness has been shown to inhibit oligodendrocyte differentiation, while softer matrices promote it^14^, these changes might be a contributing factor to the differences in remyelination potential of acute (soft) vs. chronic (stiff) demyelinated lesions. Of note, the increase in stiffness is not uniform, with many 20–40 µm wide areas exhibiting greatly increased stiffness (Fig. 4) interspersed with regions that are softer than NAWM. A similar pattern appears to be evident in MS tissue with increased ECM deposition (Fig. 5), as well as morphology and cellular composition consistent with that of chronic lesions^15^.

Variations in brain regional stiffness have been previously correlated to local myelin content both developmentally^34^ and in experimentally-induced demyelination^19^. In agreement with these findings, we found that loss of myelin in acute lesions is characterized by reduced tissue stiffness compared to uninjured brain, while remyelination correlates with its recovery to normal values. The increase in tissue stiffness observed in chronic cuprizone-induced lesions and stiff MS lesions is consistent with the increase in ECM production in our AFM-analyzed sections. Although we have yet to characterize the nature of the high-stiffness aggregates present in chronically demyelinated tissue, the involvement of reactive astrocytes and increased fibronectin deposition is the most likely explanation. Astrogliosis features prominently in the poor remyelination observed in chronic MS lesions^35,36^, and has been also shown to occur extensively in cuprizone-induced demyelination^37^. Reactive astrocytes secrete large amounts of various ECM components^38^, including CSPGs which have been shown to inhibit CNS remyelination^17,39^. Increased ECM stiffness or mechanical stimulation also promote astrocyte reactivity^40,41^, and the expression of intermediate filament proteins such as GFAP and vimentin^40,41^. Our findings of increased GFAP and vimentin levels in stiffer chronic lesions in both mouse and human tissue are consistent with these observations. In agreement with recent reports^37,42^, we also found that fibronectin immunoreactivity was significantly enhanced in the cuprizone model and was also prominent in stiffer MS lesions. Notably, transient deposition of soluble fibronectin monomer during acute demyelination phase maybe be functionally different from the formation of insoluble aggregates in chronic demyelination^42^, which have been shown to inhibit OL maturation but promote OPC proliferation^43,44^. The observed patterns of increased fibronectin deposition might also be indicative of the increased angiogenesis common in demyelinated lesions^45^. However, considering that as much as 30–46% of the total area of stiff lesions displayed a significant change in elastic modulus, it is unlikely that the contribution of vasculature alone can explain these differences. Finally, major stiffness changes can not simply be accounted for as a result of altered myelin content, since a recent study shows that while acute demyelination significantly decreases tissue stiffness, inherited myelin loss, such as in the *shiverer* mouse, does not^46^. This suggests that pathological changes in parenchymal structure, for example as a result of the immune infiltration and astrogliosis in MS, are likely the major drivers of the changes in the elastic properties of the tissue.

It should also be noted that a recent AFM study^29^ using a traumatic injury model demonstrated decreased stiffness correlated with increased astrogliosis. Although seemingly contradictory, these findings match our data on acute lysolecithin demyelination, where mechanical injury is a contributing factor. Additionally, the individual areas indented were approximately 40 µm in diameter, which is an order of magnitude lower than the resolution used in our study, thus preventing the detection of regions of increased stiffness at cellular scale and below. In fact, the patterns of tissue stiffness observed in this study underscore the importance of using methods of measurement with sufficient spatial resolution (Fig. 1). The force maps produced in this study are 90 × 90 µm, with individual measurements 9 µm apart and performed using a spherical indenter 6 µm in diameter, This resulted in individual indentation areas with a diameter of 3–4 μm, which are smaller in size than the typical cell body. Furthermore, the methodology used here does not represent the limit of the resolution achievable through AFM. We have previously used probes as small as 40 nm to measure the elastic properties of single PNS myelinated fibers^14^, a resolution sufficiently high to resolve individual ECM fibrils. However, such increased resolution imposes costs in terms of the precision required during sample preparation, as well as in terms of acquisition time. We believe that the approach described here provides a suitable compromise between resolution and speed of data capture.

Although there have been efforts to examine the mechanical properties of the brain using animal models^47–49^, a closer examination of the literature shows that the stiffness values vary by more than an order of magnitude (ranging from 0.1 to more than 10 kPa)^50,51^. This variability might be attributed to differences in the methods used: oscillatory rheology, indentation with macro- and micro-scale probes, or non-invasive magnetic resonance elastography (MRE); but also to technical factors such as indentation force and velocity, and mathematical models used to interpret the data^52^. Finally, sample preparation, age, sex and species and the post-mortem interval before sample collection can introduce further variability.

One important aspect of the mouse model used in these studies was the high reproducibility of demyelination resulting from cuprizone treatment, as well the ease of precisely locating the demyelinated area in all specimens. Although large-scale testing of MS specimens using AFM microindentation presents certain technical limitations, we found that when comparing lesions obtained from age and sex matched patients, collected at similar post-mortem intervals and located in the same brain area, clear and informative patterns emerge despite the intrinsic tissue variability. Furthermore, these patterns were comparable to those seen in acute and chronically demyelinated mouse lesions. While the initial AFM data from human tissue was obtained from thicker sections that were not optically transparent, we have since attempted AFM measurements of 100 µm cryosections of human tissue (Fig. S1). These sections were taken from adjacent (~1 mm distant) regions of two of the lesions analyzed in Fig. 5, and despite the inherent variability of pathological human tissue, exhibit elastic properties consistent with those we originally measured (Fig. S1D). Moroever, the raw stiffness values measured in the 100 µm slices were consistent with those previously measured in thicker tissue sections (Table S1), with nearly identical values observed for NAWM in both sampling conditions. These findings set the stage for future work in which we will be able to examine the relationship between MS lesion stiffness and their ECM and cellular composition in greater detail.

In conclusion, we have characterized *ex-vivo* the mechanical properties of demyelinated mouse and human brains at cellular scale using AFM microindentation. This approach identified reproducible changes in relative tissue stiffness in acute and chronic demyelination. Specifically, acute lesions are softer while chronic lesion are stiffer than uninjured white matter. While remyelination of acute soft lesions is linked to recovery of tissue stiffness to control values, enhanced ECM deposition correlates with increased stiffness in chronically demyelinated lesions. Since stiff substrates has been shown to inhibit oligodendrocyte differentiation and soft substrates to promote it^14^, our data support the notion that increased stiffness of the ECM may be an important factor contributing to remyelination failure. Although transplantation of OL progenitors is a promising therapeutic tool to promote remyelination in chronic MS, this approach can only be successful is the environment is both biochemically and mechanically permissive (“soft”) for OL survival and differentiation.

## Methods

### Lysolecithin-induced demyelination

12-week-old C57BL/6 mice were injected unilaterally into the corpus callosum (5.5 mm anterior to lambda, 1 mm lateral to bregma, 2 mm deep) with 1.5 mL of a solution of 1% lysolecithin suspended in PBS as previously described in^30^. The animals were sacrificed 7 or 21 days later. These points were selected to correspond to well-established phases of active demyelination and remyelination, respectively^53^.

### Cuprizone-induced demyelination

8-week old C57BL/6 male mice were fed commercially-prepared 0.2% cuprizone chow (Envigo Co.) for either 6 weeks, leading to acute demyelination, or 12 weeks, leading to chronic demyelination and remyelination failure, as described in^54^. Following each treatment time point, half of the animals were allowed to recover for 3 weeks on normal chow (remyelination), with a minimum of 3 brains collected per time point. Littermates fed normal chow were used as controls, and sacrificed as appropriate (6, 9, 12 and 15 weeks) along with experimental animals to provide age-matched controls.

### Tissue Preparation

For mouse tissue, animals were anaesthetized and sacrificed by transcardial perfusion of PBS and heparin. The brains were removed, and coronal sections 5 mm thick were cut, either centered on the region of the injection (lysolecithin model) or directly caudal of Bregma (cuprizone model). The sections were fixed in 4% paraformaldehyde for 16 hours, incubated for 24 hours in 30% sucrose, frozen in OCT, cryo-sectioned to a thickness of 100 µm using a Leica CM3050S cryostat, and washed extensively with PBS to remove residual cutting medium, and briefly stored at 4 °C in PBS. To prepare them for AFM measurement, tissue sections were placed in the center of a chloro/silane-treated glass-bottom dish, covered with a 22 × 22 mm cover slip treated with a hydrophobic coating (Rain-X), and adhered to the surface of the dish by the addition of an activated polyacrylamide (PA) mixture between the two glass surfaces. Following polymerization of the PA gel, the coverslips were removed under PBS. This procedure resulted in the tissue margins firmly embedded in the gel, stretching the tissue uniformly across the glass surface without the need for underlying adhesive. The sections were extensively rinsed to remove dust or chemical residues, and then inspected using a stereoscope to verify that the corpus callosum was clear of any obstructions or physical damage. In addition, while the hydrophobic coating on the glass coverslip serves to prevent the polyacrylamide mixture from flowing over the top of the tissue, a measurement of the gel at the tissue margin was performed to serve as a control against artifacts arising from the PA-mounting process. Sections directly adjacent (cranial and caudal to the one being analyzed) were preserved to provide additional material for IHC.

Fresh-frozen coronal sections of tissue from MS patients were obtained from the Rocky Mountain MS Center Tissue Bank. Preliminary pathological analysis to identify potential lesion areas was performed by Dr. George Zanazzi of the Columbia University Medical Center. Sections were thawed on ice, and lesioned areas along with a 3–5 mm margin of white matter were dissected out, producing rectangular tissue blocks with a thickness of 3–5 mm and a surface area of ~100–150 mm^2^. The blocks were then fixed in 4% paraformaldehyde for 16 hours, and for AFM measurements, were mounted in imaging dishes using cyanoacrylate adhesive. Following AFM, the tissue was incubated for 24 hours in 30% sucrose, and frozen in OCT. Several 50 µm sections were then cut from the face of each block, and used for IHC. Unlike in the animal model, the cryo-slicing was performed post-AFM to ensure stiffness measurements were obtained before the sectioning of irreplaceable human tissue of unknown internal architecture and integrity.

For the experiments described in Fig. S1, 100 µm cryosections of human tissue were prepared as described above for mouse tissue.

### Human and Animal Subjects

Vertebrate animal tissue (brains) was collected in accordance with the National Institutes of Health guidelines. All procedures were approved by Hunter College Institutional Animal Care and Use Committee.

The MS tissue experiments described here used anonymous cadaver samples. As such, they do not meet the criteria for “experiments on human subjects” as defined by the CUNY Human Research Protection Program, and were exempt from IRB review.

### AFM measurements

An MFP-3D-BIO Atomic Force Microscope (Asylum Research) was used to collect force maps from the brain tissue of both mouse and human samples. A CP CONT-PS-C (NanoAndMore.com) probe with a 6.1 μm polystyrene bead was used for all measurements. The Asylum Research GetReal calibration method was utilized for the determination of the spring constant (~0.2 N/m). Each force map sampled a 90 µm × 90 µm region in a 10 × 10 grid under fluid conditions (PBS). The trigger point was set to 25 nN with an approach velocity of 10 µm/sec. The force-indentation curves were fit to the Hertz model^55^ for spherical tips utilizing the Asylum Research Software to determine the Young’s Modulus, with an assumed Poisson’s ratio value of 0.45 for the sample. Force maps of stiffness along with individual stiffness values for each measured point were then exported from the Asylum Research Software for further analysis. Brightfield images of brain tissue for the determination of location of stiffness measurements were acquired using an inverted microscope (Zeiss Axio Observer Z1) that was used as the AFM base (LD Plan-Neofluar 5x 0.15NA objective).

In the lysolecithin model, measurements were performed in a grid spanning the entire visible lesion and a small margin of surrounding tissue, with 90 µm × 90 µm regions of 100 measurements each spaced 300 µm apart (Fig. 3A), while 390 µm × 90 µm regions were measured in the corresponding region of the contralateral corpus callosum to serve as controls. In the cuprizone model, a minimum of four (4) 90 µm × 90 µm regions with 100 individual indentations per region were initially measured per brain. The areas were uniformly spaced through the medial portion of the corpus callosum, as shown in Fig. 4A. Areas which exhibited mechanical damage or fouling resulting from sectioning (a potential source of error and artifacts in force-indentation measurements) were excluded from analysis.

For human samples, 90 µm × 90 µm regions (100 measurements per region) were analyzed in a grid pattern, with the measurements spaced 500 µm apart and covering an area extending from NAWM into the lesion spanning on average a rectangular area ~2 × 5 mm in size. Due to the extensive pathological changes in the MS tissue, some regions (that featured extreme variations in tissue height or appeared to contain macroscopic pieces of debris which interfered with the AFM probe cantilever) produced curves that could not be fitted accurately to the Hertz model and were discarded. These accounted for ~10–20% of the regions measured, with no apparent relationship to the pathological characteristics of the lesion. Due to the variability existing even between well-matched human samples and the lack of perfectly matched healthy controls, the stiffness measurements for each lesion were standardized to the average of the stiffness of the NAWM surrounding the lesion and expressed as relative values.

### IHC, Optical Imaging and Analysis

Immunohistochemistry and imaging were performed as described previously^30^. Images were captured using a Zeiss LSM 510 confocal microscope with a 25X water immersion objective. Image analysis was performed using ImageJ/Fiji. For quantitation of fluoromyelin intensity in the cuprizone model, mean gray values were measured within a manually delineated ROI corresponding to the AFM-measured region of the corpus callosum, and normalized to the mean gray values of the cortex directly dorsal to the corpus callosum. For the quantitation of fluoromyelin, vimentin and fibronectin intensity in the MS lesions, mean gray values of 25X fields were measured, and standardized to the mean gray values of the same stain/antibody in the NAWM surrounding the lesion. Adjustment of brightness and contrast was performed in some cases for the representative images shown, but without misrepresenting the data. Antibodies used in these studies included those reactive to: GFAP (Cell Signaling), CSPG (Sigma), Iba1 (Wako), vimentin (Santa Cruz) and fibronectin (Sigma). FluoroMyelin (ThermoFisher) was used for myelin membrane visualization. Secondary antibodies conjugated to rhodamine or cyanin 5 were obtained from Jackson Laboratories.

### Modeling the Effects of Spatial Resolution

A custom MATLAB (MathWorks) script was written to simulate effects of spatial resolution on stiffness measurements of tissue (available upon request). An input image (Fig. 1A, greyscale images in the top panels), which represents idealized stiffness values as a function of image pixel intensity, was segmented into various bins (Fig. 1A, overlaid red grids) representing the resolution of the measurements being taken. Within each bin the pixel intensity values were averaged and a normally distributed random value was added to this average in order to simulate tissue heterogeneity that is present in real tissue. The resulting force maps and data files were then exported to show how under-sampling the tissue stiffness values may affect results.

### Statistical Analyses

GraphPad Prism 7.03 was used to perform statistical analyses. The D’Agostino & Pearson test was employed to evaluate the normality of distributions. To evaluate the differences between two groups, a two-tailed Mann-Whitney test was used; and a Kruskal-Wallis ANOVA with post-hoc comparisons to evaluate the differences between multiple groups. In the text, * is used to indicate p < 0.05, **p < 0.01, ***p < 0.001 and ****p < 0.0001.

## Supplementary information

Supplementary Information
