# Supplementary material for: Acute and chronic demyelinated CNS lesions exhibit opposite elastic properties
Source: Sci Rep. 2019 Jan 30;9:999. doi: 10.1038/s41598-018-37745-7 (PMC6354022; doi:10.1038/s41598-018-37745-7)
Supplement: Supplementary file 1 — Supplementary Information [file 41598_2018_37745_MOESM1_ESM.pdf]

Supplementary Material

Acute and chronic demyelinated CNS lesions exhibit opposite elastic properties

Mateusz M. Urbanski [1], Matthew B. Brendel [2] and Carmen V. Melendez-Vasquez [1,3*]

[1] Hunter College, Department of Biological Sciences, New York, NY 10065, USA. [2] Molecular Cytology Core Facility, Zuckerman Research Center, Memorial Sloan Kettering Cancer Center, New York, NY 10065, USA. [3] The Graduate Center of the City University of New York, NY 10016, USA. [*]Correspondence should be addressed to Carmen V. Melendez-Vasquez (email: melendez@genectr.hunter.cuny.edu)

Supplemental Table 1. Elastic moduli of human MS brain sections

**Sample Type**		**E ± SD**
Fixed Human Brain, Thick Sections*	Chronic Lesions	9.148 ± 18.51
	Acute Lesions	1.314 ± 1.653
	NAWM	1.88 ± 2.54
Fixed Human Brain, 100µm Cryosections**	Lesion 2	3.26 ± 2.32
	Lesion 6	2.49 ± 1.83
	NAWM	1.93 ± 1.69

* n = 1932, 1782 and 2824 for the chronic lesions, acute lesions and NAWM, respectively.

** n = 980, 500 and 1000 for Lesion 2, Lesion 6 and NAWM, respectively.

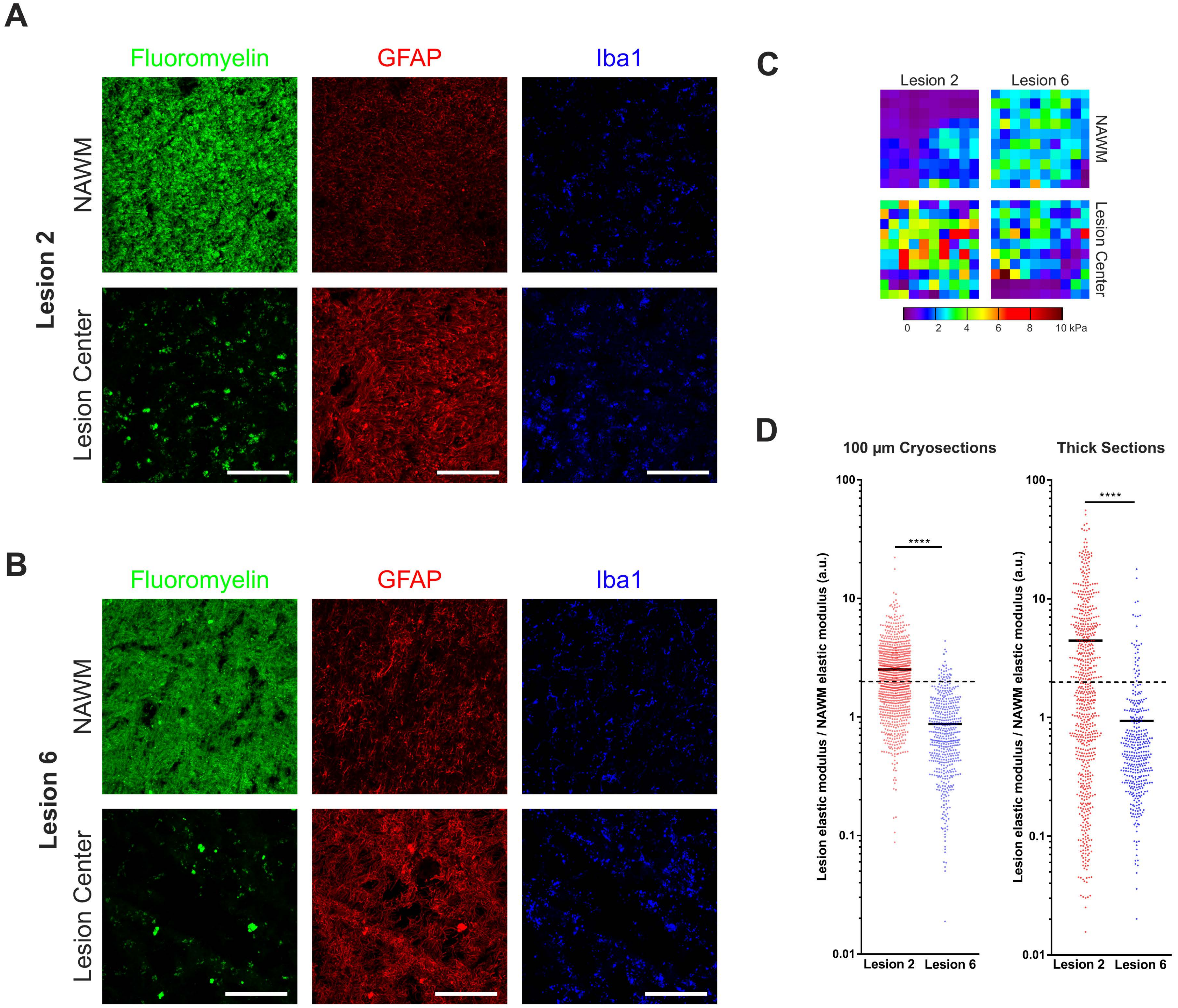

Supplemental Figure 1. AFM of 100 μm cryosections of human MS brain samples (**A-B**) Representative images showing MS lesions (stiff – lesion 2 and soft – lesion 6) and adjacent normal-appearing white matter (NAWM). The extent of myelin loss (fluoromyelin), presence of a reactive astrocytes (GFAP) and monocyte /microglia infiltration (Iba1) are shown. (Scale bars 100 μm) (**C**) Representative 90 x 90 μm AFM force maps from the lesions shown in A and B. Changes in elasticity are detectable within each lesion compared to adjacent NAWM. (**D**) Quantitation of AFM stiffness measurements from MS lesions (stiff lesion in red; soft in blue), with data from 100μm cryosections shown in the left panel, and data from the original thick sections (Fig. 5) on the right. Values are expressed as lesion stiffness relative to adjacent NAWM. Dotted line indicates two-fold increase in stiffness compared to NAWM. Bars indicate the mean value from all measurements (for 100 μm cryosections, n = 980 for lesion 2 and 500 for lesion 6; for thick sections, n = 693 for lesion 2 and 384 for lesion 6; ****$p < 0.0001$, Mann-Whitney test).
